# Preparation of Anorthite/Mullite In Situ and Phase Transformation in Porcelain

**DOI:** 10.3390/ma16041616

**Published:** 2023-02-15

**Authors:** Shao-Min Lin, Ya-Ling Yu, Ming-Feng Zhong, Huan Yang, Yang Liu, Hang Li, Chen-Yang Zhang, Zhi-Jie Zhang

**Affiliations:** 1 School of Materials Science and Engineering, Hanshan Normal University, Chaozhou 521041, China; 2 School of Materials Science and Engineering, South China University of Technology, Guangzhou 510641, China; 3 School of Materials Science and Engineering, Central South University, Changsha 410083, China; 4 Chaozhou Three-Circle (Group) Co., Ltd., Chaozhou 515646, China

**Keywords:** anorthite, mullite, phase transformation, porcelain, thermodynamic analysis

## Abstract

A high sintering temperature is required to acquire excellent performance in the production of porcelain but results in high fuel consumption. To prepare the porcelain with outstanding performance at a lower temperature, a self-produced additive containing calcium (CaK) was added into a three-component system of kaolinite–feldspar–quartz. XRD and SEM were used to characterize the samples. The toughening mechanism and Gibbs free energy were investigated. After introducing the CaK, the bending strength of the porcelain fired at 1513 K increased from 56.32 ± 0.65 MPa to 95.31 ± 0.63 MPa, which was 21.83% higher than that of the porcelain without CaK at an optimal firing temperature of 1603 K. The main crystal phase of the sample comprised mullite and quartz in the raw materials at 1453~1603 K. The anorthite was observed at 1453 K and interlocked with needle-shaped mullite at 1513 K in the porcelain after adding CaK, which resulted in the higher bending strength. Quantitative analysis indicated that the amount of anorthite decreased at 1513 K and disappeared at 1543 K; the amount of mullite increased with temperature. The Gibbs free energy of the reaction (CaO•Al_2_O_3_•2SiO_2_ + 2(Al_2_O_3_•2SiO_2_) → 3Al_2_O_3_•2SiO_2_ + CaO + 4SiO_2_) at high temperature was negative, which suggested that the formation of mullite (3Al_2_O_3_•2SiO_2_) from anorthite (CaO•Al_2_O_3_•2SiO_2_) was possible. These findings implied that the addition of CaK contributed to the appropriate phase composition and microstructure, and the excellent performance of the porcelain at a lower temperature. In addition, the transformation between anorthite and mullite was possible in the special raw material system. The results are of interest in producing anorthite/mullite ceramics at reduced sintering temperatures and the conversion between anorthite and mullite.

## 1. Introduction

The most significant development in the history of ceramics to date is the production of vitrified and translucent porcelain in ancient China [1]. The three-component system of clay–feldspar–quartz is widely used in the ceramic industry [2,3,4,5]. The main components of the sintered product are quartz (11 × 10^−6^ K^−1^), mullite (5.7 × 10^−6^ K^−1^), and feldspar glass (4.8 × 10^−6^ K^−1^). The coefficient of thermal expansion between different compositions has a significant effect on the mechanical properties and thermal stability of the ceramic products [6,7,8,9,10,11]. To form the mullite/anorthite multiphase and improve the performance of ceramics, anorthite (4.8 × 10^−6^ K^−1^) may be introduced into the sintered compact as an alternative method [12].

The preparation methods of mullite/anorthite ceramics have been investigated by a few scholars, and the formation mechanisms of mullite and anorthite are worthy of study [13,14,15]. Lin et al. prepared mullite/anorthite multiphase porous ceramics by foam injection and sintering at 1350 K~1450 K, using CaCO_3_, SiO_2_, and Al_2_O_3_ as raw materials [16]. Dong et al. used Al(OH)_3_, AlF_3_, V_2_O_5_, Suzhou kaolin, calcite, and quartz as the raw materials, to prepare the main crystalline phase of mullite/anorthite with a good interface combination via an in situ growth method at different temperatures [17]. Ilic et al. used fly ash from power plants as a raw material in ceramic production and studied the effect of firing temperature on the mullite/anorthite multiphase, and the factors that influenced the formation of anorthite/mullite multiphase ceramics were discussed [18]. At the same time, we found that although the formation temperature of anorthite and mullite varied in different systems, the variation trend of the ratio of mullite/anorthite was consistent, i.e., the ratio of mullite/anorthite increased with temperature. However, the conversion of anorthite to mullite in a low-calcium system was seldom reported and the mechanism of the above variation was not discussed further.

In the paper, a mixture of Ca_3_(PO_4_)_2_ and activated kaolinite was introduced into a three-component system of kaolinite–feldspar–quartz. The generation of an anorthite/mullite phase in situ was expected. The addition of Ca_3_(PO_4_)_2_ enhanced the formation and growth of anorthite, and the activated kaolinite was expected to enhance the formation and growth of mullite at a lower sintering temperature. The influence of anorthite/mullite on the mechanical property of the ceramic body was explored. The transformation between anorthite and mullite in the system was investigated.

## 2. Materials and Methods

### 2.1. Preparation

The raw material (Guangdong Changlong Ceramic Co., Ltd., Meizhou, China) was marked as R, and the raw material mixed with 6 wt% additive was marked as M. The additive consisted of 50 wt% activated kaolinite [19] and 50 wt% Ca_3_(PO_4_)_2_ (99%, Fuchen Chemical Reagents Factory, Tianjin, China) and was marked as CaK. A 50-g sample of the cleaned kaolinite was mixed with 2000 mL of the organic acids solutions in polypropylene bottles (2500 mL). The organic acids consisted of citric acid (20 mM) and oxalic acid (20 mM), and the solution was adjusted to pH 4. The mixtures were stirred using a magnetic stirrer, and then sealed and fully immersed in a thermostatic water-bath held at a constant temperature of 298.15 K. After reaction, the supernatants were filtered through 0.45 μm nylon filters, and the solid materials remaining were washed several times. Finally, the washed solids were dried and sieved through a 300 mesh sieve for later use.

The 50 g powders of R or M were ball milled (QM-3SP2, Nanda, Ltd., Shenzhen, China) in distilled water (200 mL) for 5 h using a polyethylene bottle (500 mL) with a zirconia ball (10 mm). The slurry was filtered through 300 mesh nylon filters and collected for analysis after being milled. After drying at 378 ± 1 K in air (DHG-9030A, Shanghai Yiheng Experimental Instrument Co., Ltd., Shanghai, China), the powders were ground in an agate mortar and passed through a 200 mesh nylon sieve. Specimens were prepared by dry-pressing molding (120 mm (L) × 20 mm (W) × 3 mm (H)) at 20 MPa at 15 MPa. In order to investigate the effect of temperature on the sample, which could be applied in the field of household porcelain, the samples were fired at different temperatures (1453 K~1603 K) in an electric furnace. At the same time, the conversion of anorthite and mullite was the focus of this research, and the specific heating rate (5 °C/min) and holding time (2 h) were used to make the reaction more adequate in this system.

### 2.2. Methods

The chemical compositions of the samples were analyzed by X-ray fluorescence spectrometry (XRF; PANalytical Axios PW4400, Raalte, The Netherlands) with a fusion dissolution technique.

The phase composition of samples was identified by an X-ray diffractometer (XRD; D/Max-IIIA, Rigaku, Tokyo, Japan), operated at 30 kV and 10 mA with Cu Kα radiation and a curved graphite secondary monochromator covering 2θ between 10 and 90° with a step width of 0.02° and 0.1 s data collection per step. The data analysis was performed using PDXL by Rigaku. The quantitative analysis was conducted by the external standard method [20,21], and Si was used as the external standard for XRD analysis.

The microstructure of the samples was measured using a scanning electron microscope (SEM; Quanta 200, FEI, Hillsboro, OR, USA) with a field emission gun, operating normally at 15–20 kV of acceleration voltage in a high vacuum environment. The sintered samples were etched by 20% (*w*/*w*) hydrofluoric acid solution for 2 min before platinum sputtering treatment.

The bulk density (g/cm^3^) of the samples was measured based on Archimedes’ principle. The water absorption (%) of the samples was obtained by the GB/T 3299-2011.

D_b_ = [m_1_/(m_2_ − m_3_)] × *ρ* (1)

W_a_ = [(m_2_ − m_1_)/m_1_] × 100% (2)

m_1_ = dry weight (g) of samples placed in the drying oven.

m_2_ = wet weight (g) of samples after complete immersion in distilled water under constant vacuum.

m_3_ = float weight (g) of samples in water.

*ρ* = density of distilled water (1 g/cm^3^).

D_b_ = bulk density (g/cm^3^).

W_a_ = water absorption (%).

The bending strength (MPa) was performed using a microcomputer control universal material test machine (TFW-100B, Jinan Xinguang Test Machine Manufacturing Co., Ltd., Jinan, China).

σf = (3 × F_max_ × L)/(2 × b × d^2^) (3)

F = maximum load (N).

L = support span (mm).

b = width of the tested beam (mm).

d = thickness of the tested beam (mm).

σf = bending strength (MPa).

Five samples from each group were measured to obtain reliable water absorption, bulk density, and bending strength values [5,22]. The weight (g) of samples was measured using a precision electronic balance (FA3204B, Tianmei, Shanghai, China) with a density determination device (MD, Tianmei, Shanghai, China).

### 2.3. Calculation

A simplified formula for calculating the Gibbs free energy with temperature during this reaction was as follows:

(4) $$\Delta G_{T}^{\theta }=\Delta H_{T_{0}}^{\theta }-T\Delta {Ф}_{T}$$

where $\Delta G_{T}^{\theta }$ is the Gibbs free energy function of the reaction at temperature *T*. If $\Delta G_{T}^{\theta }$ is negative, the reaction is forward; if $\Delta G_{T}^{\theta }$ is positive, the reaction can only reverse.

The relevant thermodynamic parameter [23] referred to the Handbook of Practical Inorganic Thermodynamics Data (second edition), and the range of the calculation temperature was 900 to 1800 K. The standard formation enthalpy ($\Delta H_{f,\;298}^{\theta }$) and Gibbs free energy (Φ) of the materials used are shown in Table 1 and Table 2, respectively.

## 3. Results and Discussion

### 3.1. Properties of the Sintered Samples

**Figure 1:**
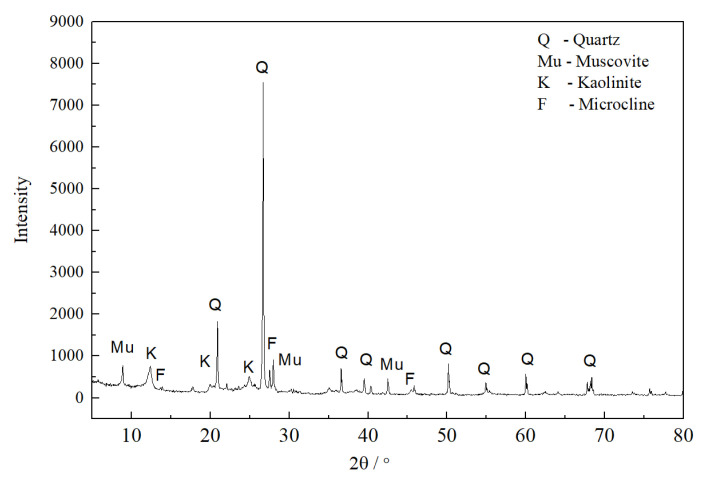
XRD pattern of the raw material.

The main crystals of the raw material were quartz (PDF#65-0466), muscovite (PDF#01-1098), kaolinite (PDF#14-0164), and microcline (PDF#22-0687) (Figure 1). Table 3 shows the chemical compositions of R and M. The raw material mainly contained silicate minerals, such as quartz, feldspar, and kaolinite. The chemical composition was mainly SiO_2_ and Al_2_O_3_. The results suggested that the raw material was prepared according to the typical three-component ceramic formula.

The water absorption, bulk density, and bending strength of the samples (R and M), are shown in Table 4. The performance of the sample improved after introducing CaK.

The water absorption of M at 1483 K was (0.39 ± 0.02)%, less than the requirement of 0.50% for daily ceramics; the water absorption of R at 1543 K was (1.17 ± 0.03)%, more than 0.50%. In the range of 1453 K~1513 K, the bulk density and bending strength of M increased with the temperature, and were higher than those of R. The bending strength of M at 1513 K was 95.31 MPa, 21.83% higher than that of R at an optimal firing temperature of 1603 K. This indicated that the addition of CaK facilitated increases in the compactness and mechanical properties of the ceramics at a lower temperature. For the raw material mixed with 6 wt% CaK, the optimal firing temperature was 1513 K. The firing temperature reduced by 90 K; the cost of the product decreased significantly. In the production of daily ceramics, the higher the firing temperature, the higher the energy consumption. According to the calculation of heat balance, if the firing temperature decreased from 1673 °C to 1573 °C, the heat consumption of the unit product could be reduced by 20%; if the firing temperature was reduced from 1573 °C to 1473 °C, the heat consumption of the unit product could be reduced by more than 11% [24].

**Figure 2:**
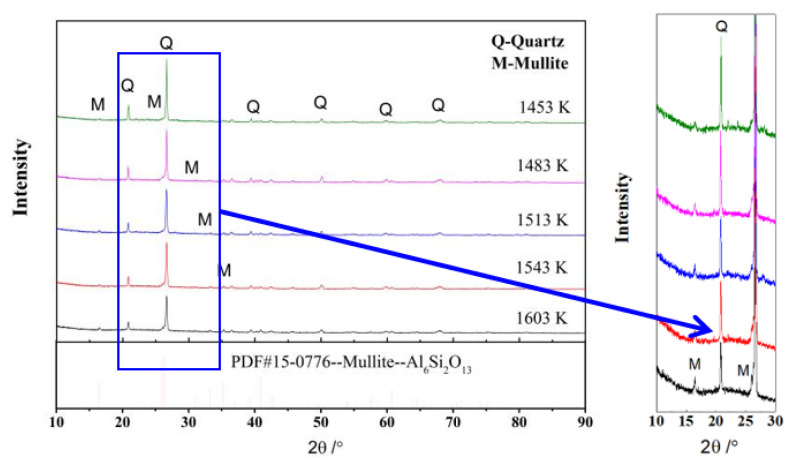
XRD patterns of R at different temperatures.

**Figure 3:**
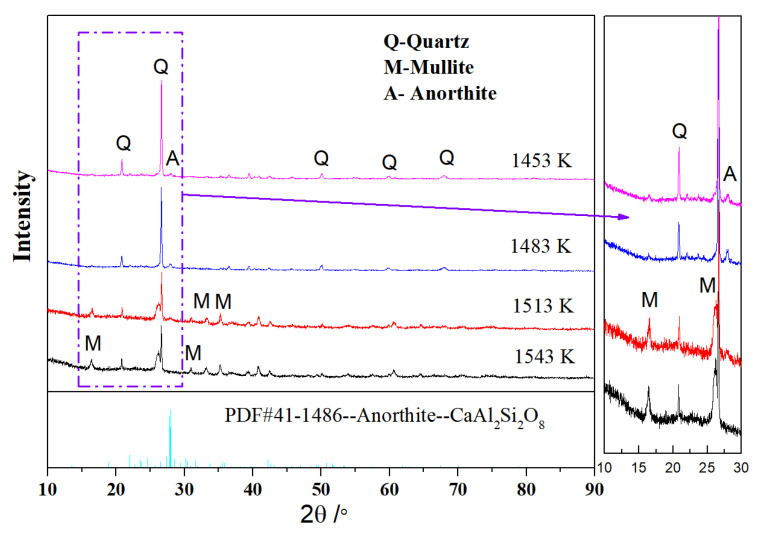
XRD patterns of M at various temperatures.

Figure 2 and Figure 3 exhibit the XRD patterns of R and M at different temperatures, respectively. The main crystal phases of the raw material were mullite and quartz (Figure 2). The new crystal phase of anorthite was observed in the XRD spectrum of M at 1453 K (Figure 3). The background peaks of M at 1513 K and 1543 K were attributed to the large amount of amorphous phase [25,26,27]. In the raw material (M) with the traditional three-component system, the XRD peak intensities of quartz decreased with the increase in temperature, while the XRD peak intensities of mullite increased with temperature, indicating that the increase in temperature enhanced the formation and growth of mullite. The reduction in the XRD peak intensities of quartz was attributed to the formation of a large amount of glass phase, which melted the quartz at high temperature. After adding additives, the changes in the XRD peak intensity of mullite and quartz were similar to that in the raw material, while the XRD peak intensities of anorthite first increased and then decreased before finally disappearing.

Quantitative analysis (Table 5) showed that the content of anorthite first increased, then decreased at 1513 K, and finally disappeared at 1543 K in M; with increasing firing temperature, the total content of mullite and anorthite in M increased, the content of quartz decreased, and the content of the glass phase increased, due to the formation of large amounts of melts and the fusion of quartz at a higher temperature. The results indicated that the addition of CaK promoted the formation and growth of anorthite/mullite. The anorthite/mullite crystals were responsible for the higher bulk density and bending strength of M at 1513 K (Table 4).

**Figure 4:**
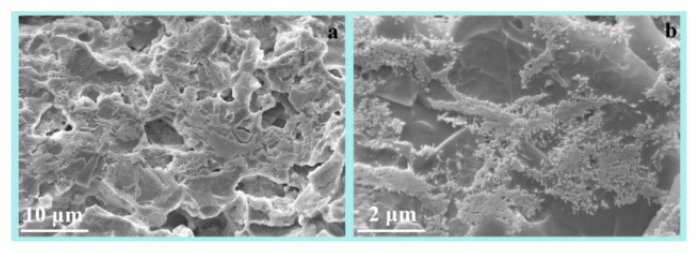
SEM images of R with different scale bar ((**a**) 10 μm; (**b**) 2 μm) at 1603 K.

**Figure 5:**
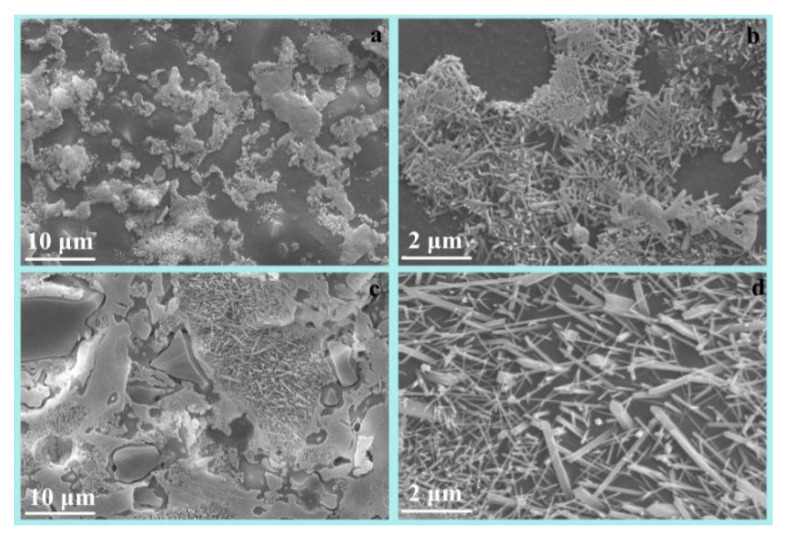
SEM images of M at 1513 K (**a**,**b**) and 1543 K (**c**,**d**).

SEM images of R and M are shown in Figure 4 and Figure 5, respectively. The microstructure of the dense sintering body consisted of crystals, glass, and pores (Figure 4a). The crystal phases of R were mullite and quartz (Figure 2), which were combined by the glass phase. The circular pore in samples would decrease the bulk density and increase the water absorption. The mullite crystal was of benefit in increasing the mechanical performance of the ceramic. The higher aspect ratio of mullite would contribute to the higher bending strength of the ceramic. The length of the mullite crystal was mostly less than 0.5 μm in R at 1603 K (Figure 4b).

Decreases were observed in the size of pore and the amount of glass phase formed in M at 1513 K (Figure 5a), compared with the morphology of R at 1603 K. At the same time, numerous tiny needle-shaped crystal were formed in M at 1513 K (Figure 5b); the interlocked crystals were identified as anorthite and mullite by XRD (Figure 3). The cross-linking anorthite/mullite crystals likely resulted in the higher bending strength of M (Table 4). When the sintering temperature increased from 1513 K to 1543 K, the anorthite crystals disappeared (Table 5) and the mullite became more stick-shaped (Figure 5d). These results suggested that mixing additives into the raw material could achieve a desired microstructure at a lower sintering temperature. The needle-shaped anorthite/mullite crystal improved the mechanical performance of the ceramics. The small reduction in the bending strength, which decreased at 1543 K (Table 4), was due to the disappearance of the anorthite crystal, the decrease in the aspect ratio of the mullite, and the increase in the glass phase; the mechanical properties of the glass phase were worse than those of the anorthite.

### 3.2. Thermodynamic Analysis of the Conversion of Anorthite and Mullite

With an increase in temperature from 1483 K to 1543 K, the content of anorthite gradually decreased until it disappeared, while the content of mullite crystals and the glass phase increased (Table 5). The results implied that the formation and growth of mullite was promoted with the reduction in anorthite at a higher temperature, which was possible due to the interconversion between anorthite and mullite in this raw material system.

In the reaction system, CaO, Al_2_O_3_, and SiO_2_ were the main components (Table 3). In order to investigate the phenomenon further, the combination reaction (Reaction 1 and Reaction 2) of anorthite and mullite was discussed thermodynamically. Anorthite was formed by CaO, Al_2_O_3_, and SiO_2_ in Reaction 1, and mullite was formed by Al_2_O_3_, and SiO_2_ in Reaction 2.

The interconversion reaction between anorthite and mullite in this raw material system was described by Reaction 3 and Reaction 4. Reaction 3 was a decomposition reaction, wherein CaO•Al_2_O_3_•2SiO_2_ was decomposed to CaO•Al_2_O_3_•2SiO_2_, CaO, and SiO_2_. Reaction 4 was a complex reaction. The metakaolinite (Al_2_O_3_•2SiO_2_) would form from kaolinite in the raw material (Figure 1) when the firing temperature was above 600 °C. Thus, reactants with CaO•Al_2_O_3_•2SiO_2_ and metakaolinite (Al_2_O_3_•2SiO_2_) were designed.

CaO + Al_2_O_3_ + 2SiO_2_ → CaO•Al_2_O_3_•2SiO_2_Reaction 13Al_2_O_3_ + 2SiO_2_ → 3Al_2_O_3_•2SiO_2_Reaction 23(CaO•Al_2_O_3_•2SiO_2_) → 3Al_2_O_3_•2SiO_2_ + 3CaO + 4SiO_2_Reaction 3CaO•Al_2_O_3_•2SiO_2_ + 2(Al_2_O_3_•2SiO_2_) → 3Al_2_O_3_•2SiO_2_ + CaO + 4SiO_2_Reaction 4

The Gibbs free energy $\Delta G_{T}^{\theta }$ of Reaction 1 and Reaction 2 are shown in Table 6. The Gibbs free energy of both reactions decreased with increasing temperature. The results indicated that the higher the temperature, the more easily the anorthite and mullite phases were formed when the reactants were binary oxide. The calculation results was consistent with the experiment of the solid phase reaction. With the increase in temperature, the activity of the reactant increased, the collision frequency of the atom increased, and the reaction more easily occurred.

Compared with the Gibbs free energy of Reaction 2, the Gibbs free energy of Reaction 1 was lower at the same temperature. This means that anorthite was preferentially formed during both reactions at the same temperature compared with mullite. The result was verified by the analysis of the phase content (Table 5). The contents of anorthite and mullite in M at 1453 K were 4.27 wt% and 19.21 wt%, respectively. When the temperature increased to 1483 K, the contents of anorthite and mullite in M at 1453 K were 7.03 wt% and 20.43 wt%. The content of anorthite increased by 64.6%, while the content of mullite increased by 6.4%, when the temperature was increased by 30 K.

The possibility of phase transformation between anorthite and mullite was studied by Reaction 3 and Reaction 4. Reaction 3 was a decomposition reaction, and the reactant was anorthite. The Gibbs free energy of Reaction 3 was positive (Table 7) at 1300~1800 K, which implied that Reaction 3 could not happen spontaneously.

In Reaction 4, anorthite and Al_2_O_3_•2SiO_2_ were the reactants, and mullite CaO and SiO_2_ were the products. The Gibbs free energy of Reaction 4 was negative (Table 7) at 1300~1800 K, which suggested that the conversion of anorthite to mullite was feasible in this system.

Al_2_O_3_ and SiO_2_ were abundant in the sample of M (Table 3), and the formation of amorphous Al_2_O_3_•2SiO_2_ at the temperature of 1483–1543 K was possible. At the same time, residual metakaolinite likely participated in the reaction. Thus, the interconversion between anorthite and mullite was able to occur. The change in the contents of anorthite and mullite in M from 1513 K to 1543 K confirmed the analysis of the calculation and led to another interesting phenomenon. The content of anorthite reduced by 4.16 wt%, and the content of mullite increased by 5.3 wt%. If the anorthite converted to mullite completely according to Reaction 4, the content of mullite should be 6.4 wt%. The value in the experiment was less than that in theory. The gap between the experimental and the theoretical likely resulted from the lack of Al_2_O_3_•2SiO_2_ or the unknown reaction path of anorthite. The subject is worthy of further study.

## 4. Conclusions

A mixture of calcium phosphate and activated kaolin (CaK) was introduced into a three-component system of kaolinite–feldspar–quartz, and the anorthite/mullite crystals were prepared in the porcelain. After the introduction of CaK, the water absorption of M was (0.39 ± 0.02)% at 1483 K, less than the requirement for daily ceramics of 0.50%, while the water absorption of R at 1483 K was (3.17 ± 0.06)%. The bending strength of M at 1513 K was 95.31 MPa, 21.83% higher than that of R at an optimal firing temperature of 1603 K. XRD analyses showed that anorthite was observed in the XRD spectrum of M at 1453 K. The main crystal phases of M were anorthite, mullite, and quartz. The interlocked needle-shaped crystal of anorthite and mullite with a high aspect ratio was confirmed by SEM images and XRD results, while the length of mullite in R at 1603 K was less than 0.5 μm. These indicated that the addition of CaK optimized the phase composition and the microstructure of the porcelain body, improving the performance of the porcelain at a lower temperature. With increasing temperature, quantitative analysis showed that the content of anorthite first increased, then decreased at 1513 K, and finally disappeared at 1543 K; the content of mullite and the glass phase increased. The results suggested that the conversion between anorthite and mullite may have occurred.

Thermodynamic analysis revealed that the Gibbs free energy of the formation of anorthite was lower than that of mullite at the same temperature. This meant that anorthite was preferentially formed, compared with mullite. The content changes in anorthite and mullite verified the calculation results. The increment of anorthite was higher than that of mullite, when the temperature increased from 1453 K to 1483 K. When Al_2_O_3_•2SiO_2_ was the reactant, it was possible that the mullite was formed from anorthite. These data implied that anorthite formed at a lower temperature with the addition of CaK; and the anorthite could transform into mullite at an elevated temperature in a special raw material system. At the same time, the gap between the experimental data and the calculation data of the content changes for anorthite and mullite warrants further investigation. The results are of interest in producing anorthite/mullite ceramics at reduced sintering temperatures and the conversion between anorthite and mullite.

## Figures and Tables

**Table 1:** Standard formation enthalpy ($\Delta H_{f,\;298}^{\theta }$/J·mol^−1^) of the substance at different temperatures.

The Substance	Al_2_O_3_	SiO_2_	CaO	CaO•Al_2_O_3_•2SiO_2_	3Al_2_O_3_•2SiO_2_	Al_2_O_3_•2SiO_2_
$\Delta H_{f,\;298}^{\theta }$/J·mol^−1^	−1,675,274	−908,346	−634,294	−4,222,493	−6,819,209	−3,211,220

**Table 2:** Gibbs free energy (Φ/J·mol^−1^·K^−1^) of the substance at different temperatures.

*T*/K	Al_2_O_3_	SiO_2_	CaO	CaO•Al_2_O_3_•2SiO_2_	3Al_2_O_3_•2SiO_2_	Al_2_O_3_•2SiO_2_
1300	124.10	85.54	74.02	382.57	574.53	306.95
1400	130.82	89.36	77.00	399.84	602.12	321.74
1500	137.27	93.01	79.85	416.48	628.59	335.90
1600	143.46	96.50	82.57	432.52	653.99	349.47
1700	149.42	99.86	85.18	448.00	678.42	362.50
1800	155.15	103.08	87.69	462.96	701.91	375.04

**Table 3:** Chemical composition (wt%) of R and M.

Samples	SiO_2_	Al_2_O_3_	Fe_2_O_3_	MgO	CaO	Na_2_O	K_2_O	TiO_2_	MnO	P_2_O_5_	Others	LOI ^1^
R	68.38	26.18	1.15	0.09	0.05	0.26	3.55	0.08	0.12	0.02	0.12	8.95
M	66.01	25.74	1.12	0.09	1.67	0.25	3.41	0.08	0.12	1.39	0.12	8.91

^1^ Loss on ignition.

**Table 4:** Water absorption (%), buck density (g/cm^3^), and bending strength (MPa) of R and M at different temperatures.

Sample	R ^1^					M ^2^			
	1453 K	1483 K	1513 K	1543 K	1603 K	1453 K	1483 K	1513 K	1543 K
Water absorption/%	8.63 ± 0.11	3.17 ± 0.06	1.63 ± 0.09	1.17 ± 0.03	0.36 ± 0.02	1.84 ± 0.07	0.39 ± 0.02	0.22 ± 0.01	0.21 ± 0.04
Bulk density/(g·cm^−3^)	1.88 ± 0.03	2.05 ± 0.05	2.17 ± 0.09	2.26 ± 0.13	2.39 ± 0.43	2.11 ± 0.06	2.43 ± 0.08	2.44 ± 0.12	2.42 ± 0.10
Bending strength/MPa	39.15 ± 1.07	48.14 ± 0.84	56.32 ± 0.65	69.16 ± 0.73	78.23 ± 0.76	63.43 ± 0.98	74.38 ± 0.55	95.31 ± 0.63	90.17 ± 0.47

^1^ Raw material. ^2^ Raw material mixed with 6 wt% CaK.

**Table 5:** Content (wt%) of phase for M at different temperatures.

Temperature/K	Anorthite	Mullite	Quartz	Amorphous Phase	The Sum of Anorthite and Mullite
1453	4.27	19.21	31.28	45.24	23.48
1483	7.03	20.43	24.23	48.31	27.46
1513	4.16	27.23	15.17	53.44	31.39
1543	-	32.53	11.86	55.61	32.53

**Table 6:** Gibbs free energy $\Delta G_{T}^{\theta }$ of Reaction 1 and Reaction 2.

*T*/K	Reaction 1			Reaction 2		
	$\Delta H_{298}^{\theta }$ (J·mol^−1^)	ΔΦ (J·mol^−1^·K^−1^)	$\Delta G_{T}^{\theta }$ (J·mol^−1^)	$\Delta H_{298}^{\theta }$ (J·mol^−1^)	ΔΦ (J·mol^−1^·K^−1^)	$\Delta G_{T}^{\theta }$ (J·mol^−1^)
1300	−96,233	13.37	−113,614	23,305	31.15	−17,190
1400		13.3	−114,853		30.94	−20,011
1500		13.34	−116,243		30.76	−22,835
1600		13.49	−117,817		30.61	−25,671
1700		13.68	−119,489		30.44	−28,443
1800		13.96	−121,361		30.30	−31,235

**Table 7:** Gibbs free energy·$\Delta G_{T}^{\theta }\;$ of Reaction 3 and Reaction 4.

*T*/K	Reaction 3			Reaction 4		
	$\Delta H_{298}^{\theta }$ (J·mol^−1^)	ΔΦ (J·mol^−1^·K^−1^)	$\Delta G_{T}^{\theta }$ (J·mol^−1^)	$\Delta H_{298}^{\theta }$ (J·mol^−1^)	ΔΦ (J·mol^−1^·K^−1^)	$\Delta G_{T}^{\theta }$ (J·mol^−1^)
1300	301,960	−8.96	313,609	−441,954	−5.76	−434,466
1400		−8.98	314,538		−6.76	−432,490
1500		−9.29	315,891		−7.80	−430,254
1600		−9.85	317,714		−8.90	−427,714
1700		−10.62	320,011		−9.96	−425,022
1800		−11.58	322,811		−11.12	−421,938

## Data Availability

Not applicable.
